# Modified substrate specificity of a methyltransferase domain by protein insertion into an adenylation domain of the bassianolide synthetase

**DOI:** 10.1186/s13036-019-0195-y

**Published:** 2019-07-31

**Authors:** Fuchao Xu, Russell Butler, Kyle May, Megi Rexhepaj, Dayu Yu, Jiachen Zi, Yi Chen, Yonghong Liang, Jia Zeng, Joan Hevel, Jixun Zhan

**Affiliations:** 1 Department of Biological Engineering, Utah State University, 4105 Old Main Hill, Logan, UT 84322-4105 USA; 2 Department of Chemistry and Biochemistry, Utah State University, 0300 Old Main Hill, Logan, UT 84322-0300 USA; 3 Department of Applied Chemistry and Biological Engineering, College of Chemical Engineering, Northeast Electric Power University, Jilin, 132012 Jilin China; 4 Key Laboratory of Modern Preparation of TCM, Ministry of Education, Jiangxi University of Traditional Chinese Medicine, Nanchang, 330004 Jiangxi China

**Keywords:** Nonribosomal peptide synthetase, Methyltransferase domain, Substrate specificity, Domain removal, Heterologous expression, In vitro reaction

## Abstract

**Background:**

Creating designer molecules using a combination of select domains from polyketide synthases and/or nonribosomal peptide synthetases (NRPS) continues to be a synthetic goal. However, an incomplete understanding of how protein-protein interactions and dynamics affect each of the domain functions stands as a major obstacle in the field. Of particular interest is understanding the basis for a class of methyltransferase domains (MT) that are found embedded within the adenylation domain (A) of fungal NRPS systems instead of in an end-to-end architecture.

**Results:**

The MT domain from bassianolide synthetase (BSLS) was removed and the truncated enzyme BSLS-ΔMT was recombinantly expressed. The biosynthesis of bassianolide was abolished and *N*-desmethylbassianolide was produced in low yields. Co-expression of BSLS-ΔMT with standalone MT did not recover bassianolide biosynthesis. In order to address the functional implications of the protein insertion, we characterized the *N*-methyltransferase activity of the MT domain as both the isolated domain (MT_BSLS_) and as part of the full NRPS megaenzyme. Surprisingly, the MT_BSLS_ construct demonstrated a relaxed substrate specificity and preferentially methylated an amino acid (L-Phe-SNAC) that is rarely incorporated into the final product. By testing the preference of a series of MT constructs (BSLS, MT_BSLS_, cMT, XLcMT, and aMT) to L-Phe-SNAC and L-Leu-SNAC, we further showed that restricting and/or fixing the termini of the MT_BSLS_ by crosslinking or embedding the MT within an A domain narrowed the substrate specificity of the methyltransferase toward L-Leu-SNAC, the preferred substrate for the BSLS megaenzyme.

**Conclusions:**

The embedding of MT into the A2 domain of BSLS is not required for the product assembly, but is critical for the overall yields of the final products. The substrate specificity of MT is significantly affected by the protein context within which it is present. While A domains are known to be responsible for selecting and activating the biosynthetic precursors for NRPS systems, our results suggest that embedding the MT acts as a secondary gatekeeper for the assembly line. This work thus provides new insights into the embedded MT domain in NRPSs, which will facilitate further engineering of this type of biosynthetic machinery to create structural diversity in natural products.

**Electronic supplementary material:**

The online version of this article (10.1186/s13036-019-0195-y) contains supplementary material, which is available to authorized users.

## Introduction

Modular enzymes are widely involved in the biosynthesis of many biologically important molecules including both primary (fatty acids) and secondary metabolites (natural products). Natural product megasynthetases, which include polyketide synthases (PKSs), nonribosomal synthetases (NRPSs), and hybrid forms, typically combine a series of catalytic domains – often as a single polypeptide – to assemble simple molecules into a variety of structurally and functionally diverse molecules [1]. NRPSs contain three essential core domains for chain elongation, including adenylation (A), thiolation (T or PCP) and condensation (C) domains, which are combined end-on-end to form independent modules. The precursor molecule for each module is selected by an A domain, and activated for transfer to a T domain. Thus, A domains are thought to function as the primary gate keepers for precursor selection. C domains condense two units of activated precursors to form the corresponding amide or ether bonds for downstream chain elongation. Additional tailoring domains may also be present, such as epimerization (E), thioesterase (TE), and methyltransferase (MT) domains, each of which contributes to the chemical diversity and bioactivity of natural products [2]. Coordinated communication between each of these catalytic domains is essential, but the molecular mechanisms underlying this process are poorly understood.

A unique feature of MT-containing NRPSs is that the MT domains are embedded within the A domains rather than being located adjacent to independent domains [3]. The rationale for this placement in NRPSs is unknown but suggests that the MT may undergo significant motions during natural product synthesis. A recent structure of an embedded NRPS MT supports this hypothesis [4]. Furthermore, the general observation that protein domain insertions are often associated with a gain-in-function of switch-like behavior [5, 6] makes for the intriguing possibility that the MT takes on a much larger role in natural product synthesis than simple methyl transfer. Embedding the MT into the A domain may act as scaffolding or may modulate substrate specificity, activity and interdomain communication.

**Fig. 1:**
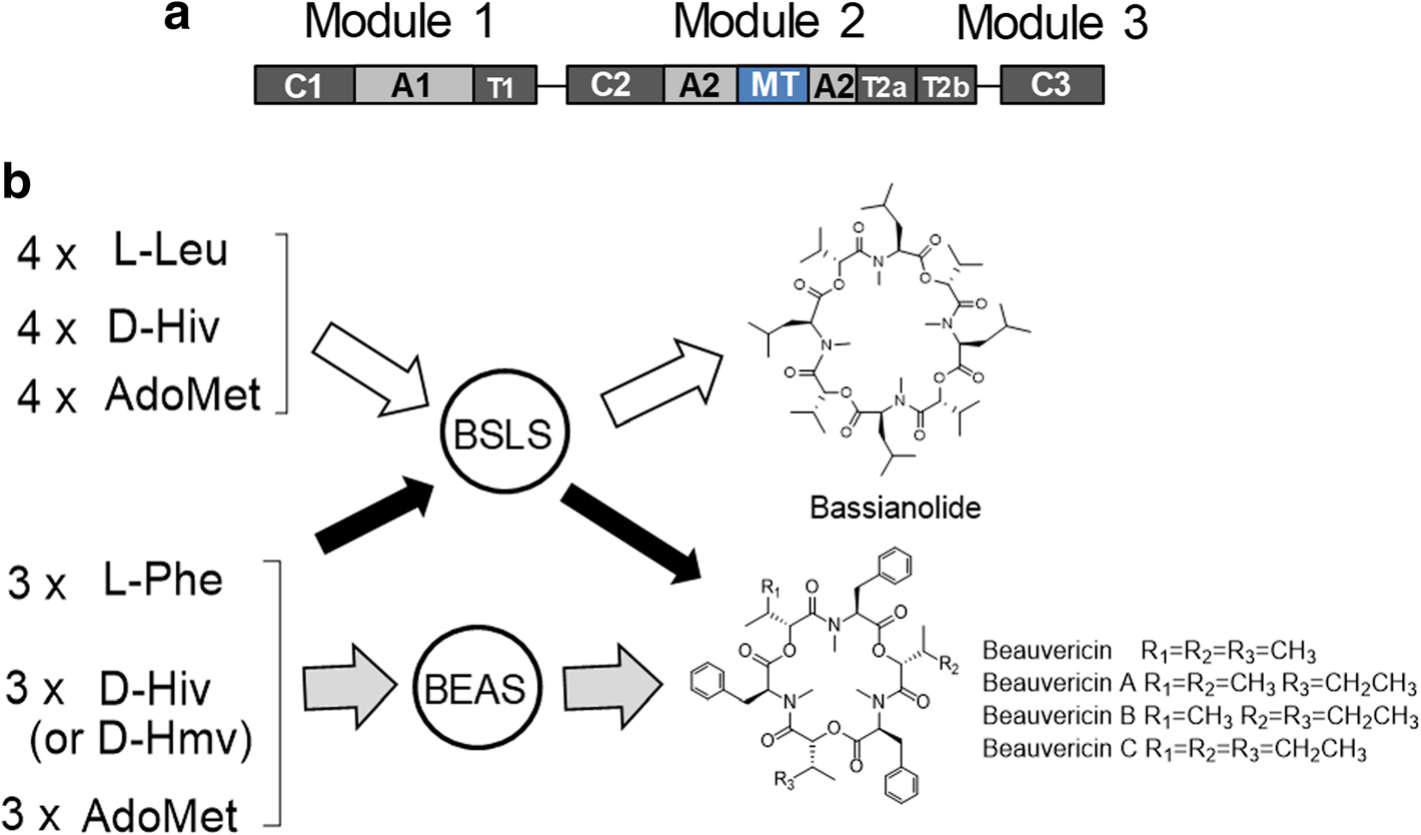
Domain organization of BSLS and BEAS and their corresponding products. **a** Domain architecture of BSLS. **b** Biosynthesis of bassianolide and beauvericins by BSLS and/or BEAS

One such embedded MT exists in BSLS, the fungal NRPS that synthesizes the cyclic octadepsipeptide bassianolide. The modular BSLS enzyme (C1-A1-T1-C2-A2-MT (embedded in A2)-T2a-T2b-C3, Fig. 1) can be found in several fungi such as *Beauveria bassiana* [7]. *B. bassiana* also produces a cyclic hexadepsipeptide, beauvericin, and the corresponding beauvericin synthetase (BEAS) has the same domain organization (Fig. 1a) [8]. Bassianolide and beauvericin have shown anticancer activities and are synthesized from amino acid and hydroxyl acid precursors [9, 10]. We have previously investigated the three C domains and the twin T2 domains in these NRPSs, and revealed that this type of NRPS uses an “alternative incorporation” approach to extend the depsipeptide chain and C3 plays a role of decision maker in the chain length control [11]. Interestingly, BSLS is flexible with both the amino acid and hydroxyl acid precursors; i.e., it produces bassianolide as the major product (containing *N*-methyl-L-Leu), together with small amounts of beauvericin and its analogs beauvericins A-C (containing *N*-methyl-L-Phe) (Fig. 1b). By contrast, BEAS is strict with the amino acid precursor and only has some flexibility with hydroxyl acid units, D-2-hydroxyisovalerate (D-Hiv) and D-2-hydroxy-3-methylvalerate (D-Hmv), to yield beauvericins (Fig. 1b). This finding suggests that the MT, as well as other catalytic domains in BSLS, have relatively relaxed substrate specificities. Therefore, BSLS was selected in this work to understand the role of the embedded MT and how its substrate specificity may be affected by the protein insertion. Our results demonstrate that the presence of a MT domain in BSLS is not essential for the product assembly, but is critical for the overall yields of the final products. Moreover, embedding the MT in the BSLS NRPS is used to tune the substrate specificity of the MT and thus functions as a secondary substrate gate keeper. This work thus provides new insights into the embedded MT domain in NRPSs, which will facilitate further engineering of this type of biosynthetic machinery to create structural diversity in natural products.

## Results

### A functional MT domain is not essential for NRP assembly

**Fig. 2:**
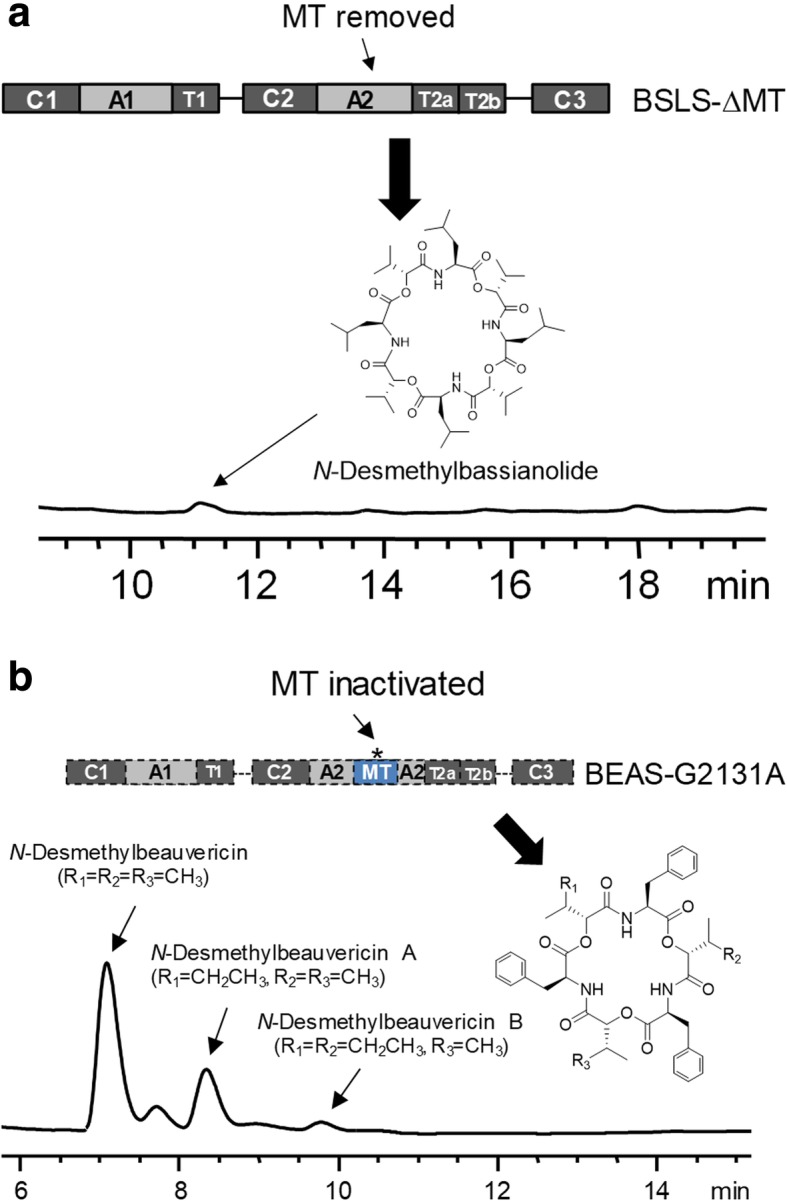
Biosynthesis of *N*-desmethylbassianolide (**a**) by BSLS-ΔMT (MT removed) and *N*-desmethylbeauvericins (**b**) by BEAS-G2131A (MT inactivated)

To examine the structural necessity of the MT domain within BSLS, we constructed BSLS-ΔMT through splicing by overlap extension (SOE) PCR and tested whether nonribosomal peptide (NRP) products can still be synthesized without the *N*-MT domain. Both BSLS and BEAS were functionally expressed in *Saccharomyces cerevisiae* BJ5464-NpgA in our previous work to yield their corresponding products [12]. Therefore, this strain was used to express BSLS-ΔMT for product analysis. LC-MS analysis revealed that the engineered enzyme BSLS-ΔMT yielded no bassianolide, but a more polar product (Fig. 2a). ESI-MS of this new product showed several ion peaks, including [M + H]^+^ at *m/z* 853.4, [M + NH_4_]^+^ at *m/z* 870.5, [M + Na]^+^ at *m/z* 875.4, and [M + K]^+^ at *m/z* 891.4 (Additional file 1: Figure S1), indicating that this product has a molecular weight of 852. This is 56 mass unis (equivalent to four CH_2_ groups) less than bassianolide, suggesting that this compound is *N*-desmethylbassianolide (Fig. 2a).

Due to the low yield of this nonmethylated product, we did not purify it for NMR analysis. Considering the yield of beauvericin and its analogs (beauvericins A-C) produced by BEAS in yeast is much higher than bassianolide, we rationalized that it might be easier to generate sufficient amounts of demethylated derivative of beauvericin. We also wanted to distinguish if the low product yields were a result of the absence of the MT domain or the absence of MT activity. To this end, we used a different approach to examine the MT domain in BEAS. Based on the alignment of BEAS with other reported MTs, it was found that the MT domain of BEAS contains a G_2129_TGTG_2133_ motif, which is consistent with the consensus GxGxG sequence that is a part of the *S*-adenosyl methionine (AdoMet) binding site [13]. Therefore, we mutated the middle G residue at position 2131 to interrupt the binding of AdoMet. We constructed BEAS-G2131A (pDY170) in a yeast expression vector and expressed it in *S. cerevisiae* BJ5464-NpgA. As shown in Fig. 2b, three new products, but not beauvericins, were formed by this mutant. Their molecular weights were determined to be 741, 755 and 768, respectively, based on the ESI-MS spectra (Additional file 1: Figure S1), corresponding to the demethylated derivatives of beauvericin, beauvericin A and beauvericin B. The major product at 7.1 min was isolated for NMR analysis, which confirmed that this product is *N*-desmethylbheauvericin (Additional file 1: Figures S2-S4) and that G2131 is a critical AdoMet binding site residue. We next constructed a yeast expression plasmid (pFC31) that only harbors the MT domain of BSLS. Co-expression of the isolated MT_BSLS_ with BSLS-ΔMT to induce *trans* methylation yielded only the demethylated product (Additional file 1: Figure S5A). Similarly, co-expression of MT_BEAS_ (pDY268) with BEAS-G2131A in the yeast did not recover the biosynthesis of beauvericins (Additional file 1: Figure S5B), suggesting that natural embedding of a functional MT domain in A2 is required to form the methylated NRPs.

### Heterologous expression and functional characterization of the MT domain from BSLS

**Fig. 3:**
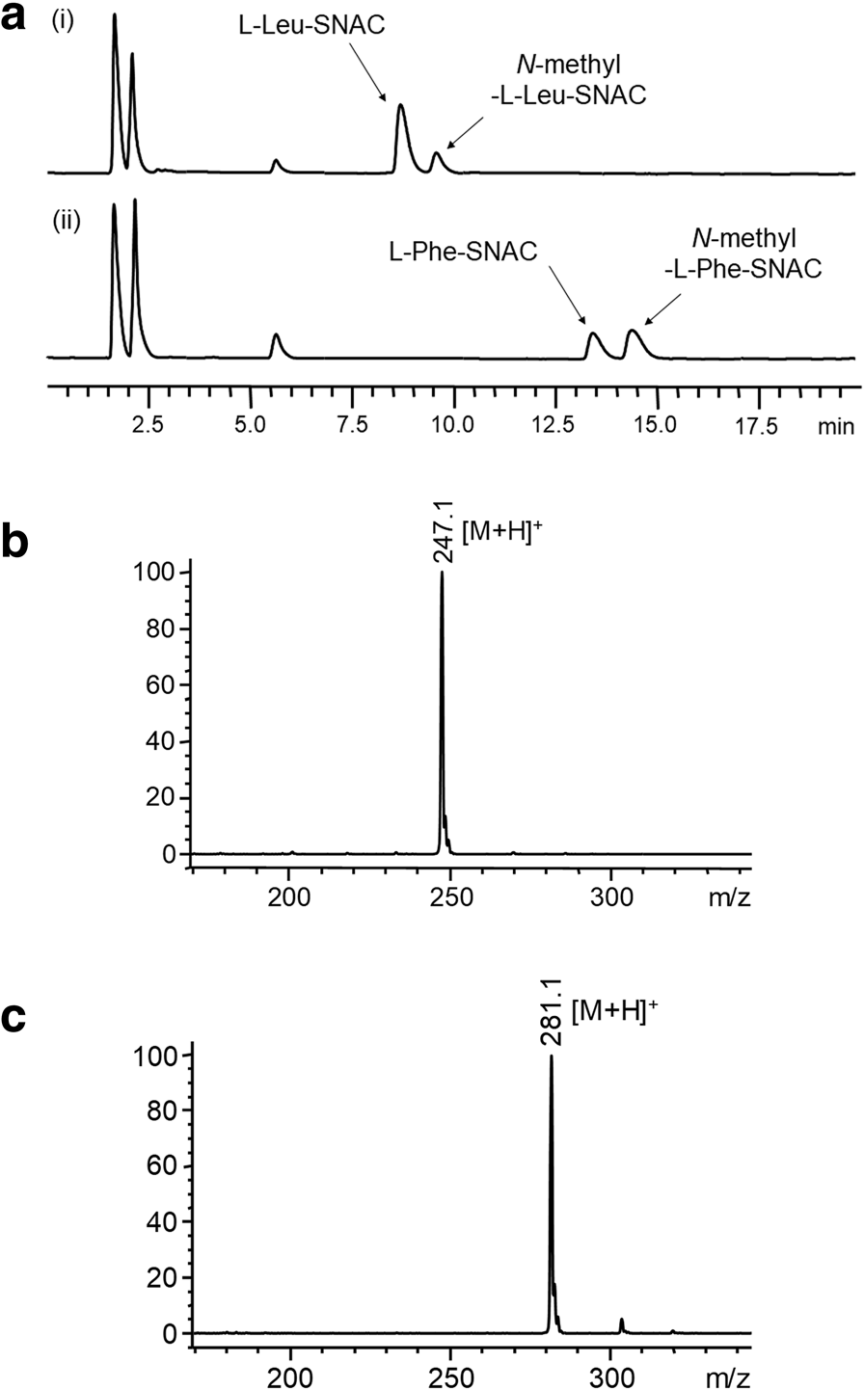
In vitro reactions of MT_BSLS_ with aminoacyl-SNACs. **a** Methylation of L-Leu-SNAC and L-Phe-SNAC by MT_BSLS_ in the presence of AdoMet. (i) L-Leu-SNAC + MT_BSLS_; (ii) L-Phe-SNAC + MT_BSLS_. **b** ESI-MS (+) spectrum of *N*-methyl-L-Leu-SNAC. **c** ESI-MS (+) spectrum of *N*-methyl-L-Phe-SNAC

In order to determine whether embedding the MT domain into the A2 domain affects MT activity, we sought to compare the methyltransferase activity of the isolated MT domain (MT_BSLS_) to the methyltransferase activity of the full megaenzyme (BSLS). The gene fragment encoding the MT domain in BSLS was cloned from the genomic DNA of *B. bassiana* into a pET28a expression vector. The MT domain was expressed in *E. coli* BL21-CodonPlus (DE3)-RIL strain (hereafter referred to as RIL) cells as a His_6_-tagged protein and was purified using Ni-NTA resin. The methyltransferase activity of the isolated MT domain (MT_BSLS_) was directly assessed using the synthetic substrate mimic aminoacyl-*N*-acetylcysteamine thioesters (aminoacyl-SNACs) to avoid the need for the A or T domains [14]. Because bassianolide contains *N*-methyl-L-Leu, L-Leu-T2a (or -T2b) is believed to be the natural substrates. Thus, we synthesized L-Leu-SNAC as a mimicking substrate. HPLC analysis showed that incubation of MT_BSLS_ with AdoMet and L-Leu-SNAC led to the formation of a new peak in the chromatogram (Fig. 3a). ESI-MS spectrum (Fig. 3b) indicated that this product has a molecular weight of 246, which is 14 mass units more than the substrate and consistent with *N*-methyl-L-Leu-SNAC. These results show that the isolated MT domain from BSLS is active with its cognate substrate. In our previous study [15], we found that BSLS can also synthesize beauvericin and its analogs beauvericins A-C when it was expressed in *S. cerevisiae* BJ5464-NpgA, suggesting that MT_BSLS_ can also methylate L-Phe-SNAC. Reaction of MT_BSLS_ with L-Phe-SNAC indeed gave rise to *N*-methyl-L-Phe-SNAC (trace ii, Fig. 3a), which was confirmed by its ESI-MS spectrum (Fig. 3c).

### Substrate specificity of the isolated MT domain of BSLS

**Fig. 4:**
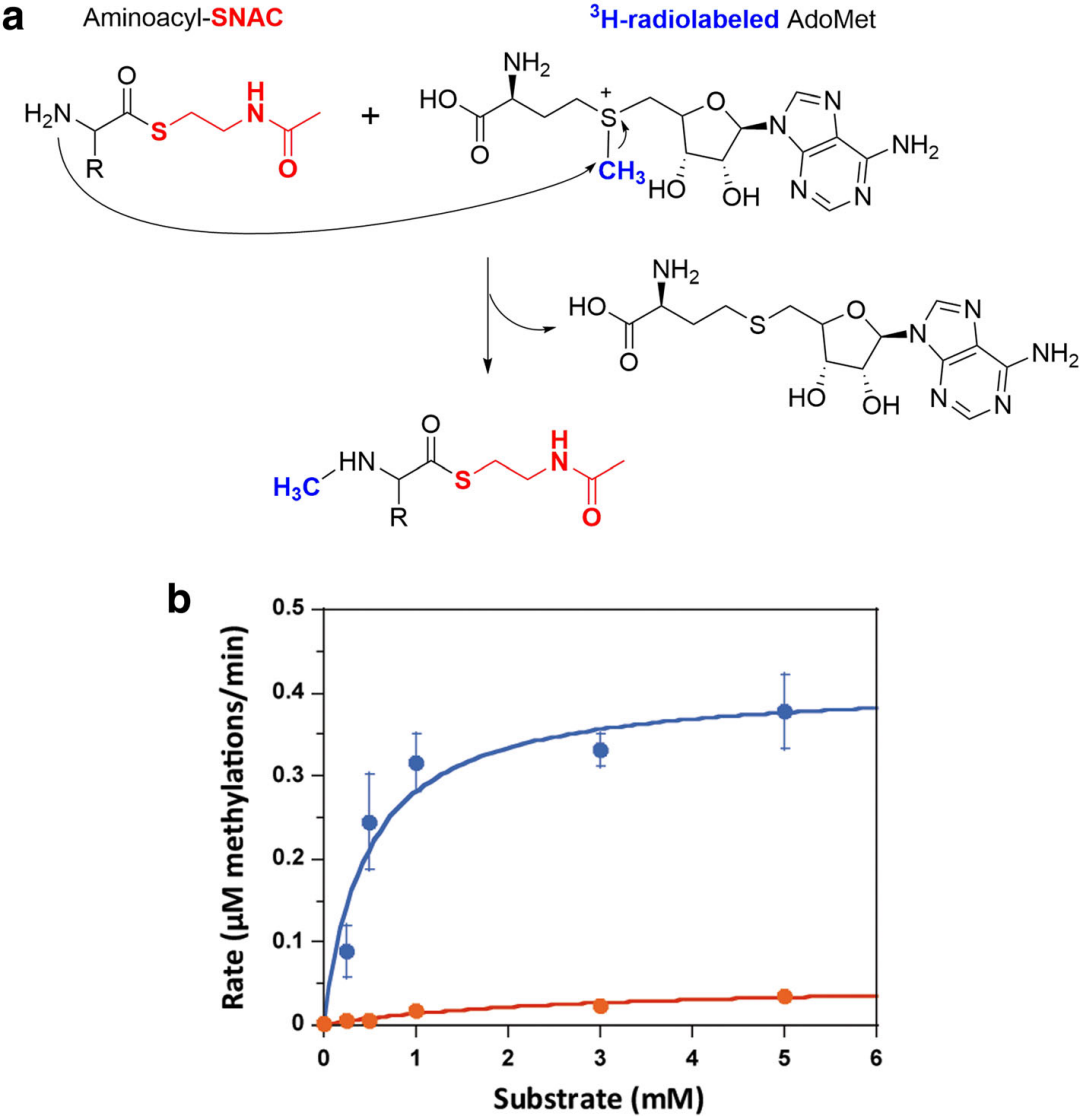
In vitro methylation activity and substrate specificity of MT_BSLS_. **a** Methylation was tracked using a ^3^H-labeled methyl group (highlighted in blue) from the co-substrate AdoMet. “R” represents amino acid side chain. **b** Concentration-dependent methylation rate of L-Phe-SNAC (blue trace) and L-Leu-SNAC (orange trace) by isolated MT_BSLS_

Given the apparent flexibility of MT_BSLS_ to use both L-Phe-SNAC and L-Leu-SNAC as substrates, we sought to determine how broad the substrate specificity of MT_BSLS_ is. Therefore, we synthesized two additional aminoacyl-SNACs, L-Ile-SNAC and L-Val-SNAC, for in vitro assays of MT_BSLS_. However, only a tiny portion of substrates were converted into *N*-methylated products, which were detected by ESI-MS analysis (Additional file 1: Figure S6A and B). In order to determine whether MT_BSLS_ displayed a preference for L-Leu-SNAC or L-Phe-SNAC, we measured the rate of methylation of these two substrates as a function of substrate concentration. The methylation assay utilized radiolabeled AdoMet (^3^H-AdoMet) as the methyl group donor in order to quantify the levels of methyl groups transferred (Fig. 4a). Interestingly, even though BSLS predominantly synthesizes bassianolide that contains the *N*-methyl-L-Leu moiety [12, 15], the isolated MT_BSLS_ enzyme was observed to possess a much higher rate of methylation with L-Phe-SNAC (V_max_ of 0.41 ± 0.040 μM/min) than with L-Leu-SNAC (V_max_ of 0.05 ± 0.01 μM/min) and a smaller K_m_ (0.46 ± 0.17 μM for Phe-SNAC and 2.8 ± 1.3 μM for Leu-SNAC) (Fig. 4b).

### The substrate specificity of MT_BSLS_ is affected by protein context

**Fig. 5:**
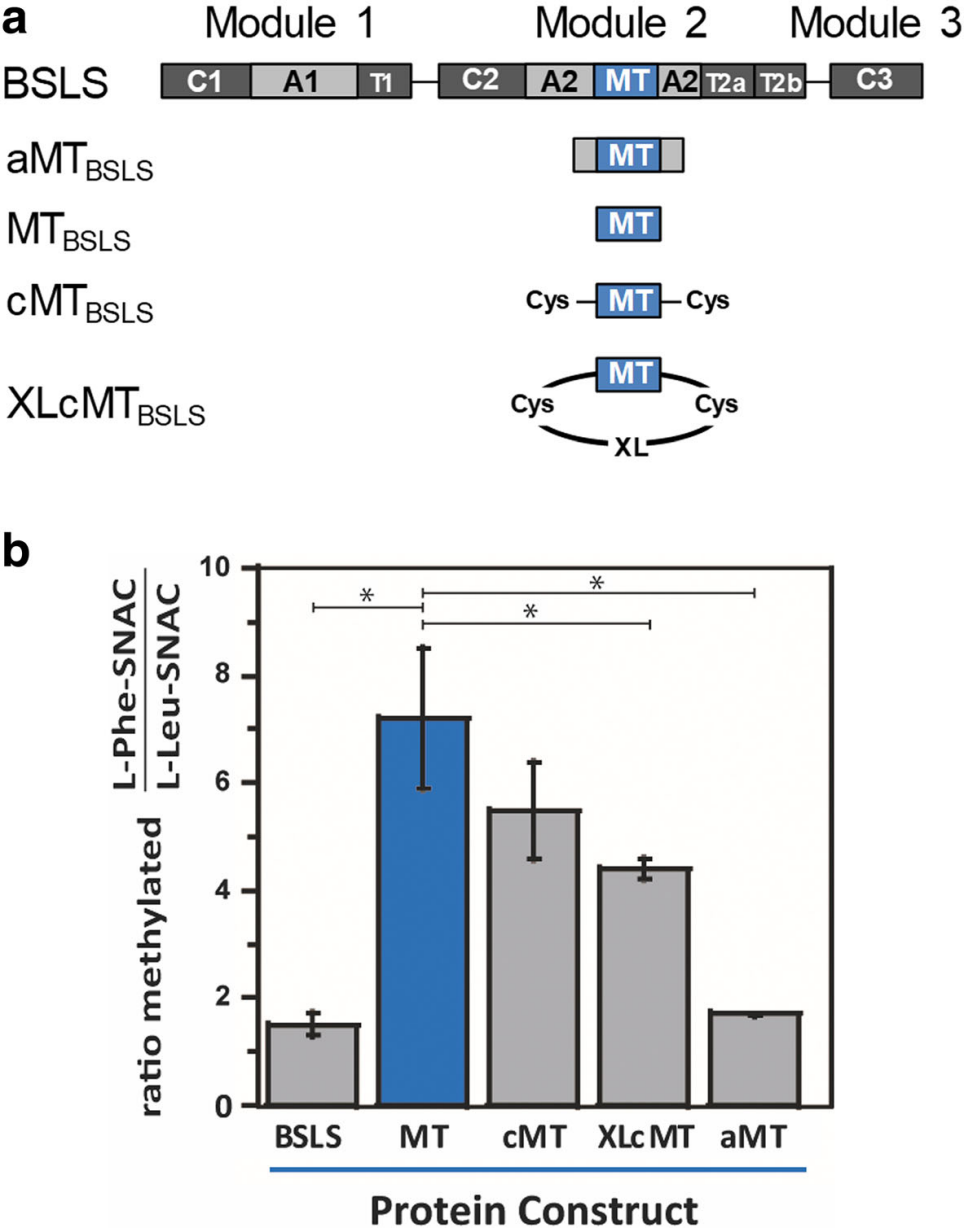
Substrate specificity of MT_BSLS_ constructs. **a** Various MT_BSLS_ constructs that were evaluated. **b** Comparison of the preference of different MT_BSLS_ constructs to L-Phe-SNAC and L-Leu-SNAC. The ratio of methylated products formed from 5 mM L-Phe-SNAC or 5 mM L-Leu-SNAC over a 10-min reaction time are plotted. The data for cMT and XLcMT represent two separate preparations of crosslinked protein, assayed in duplicate

**Fig. 6:**
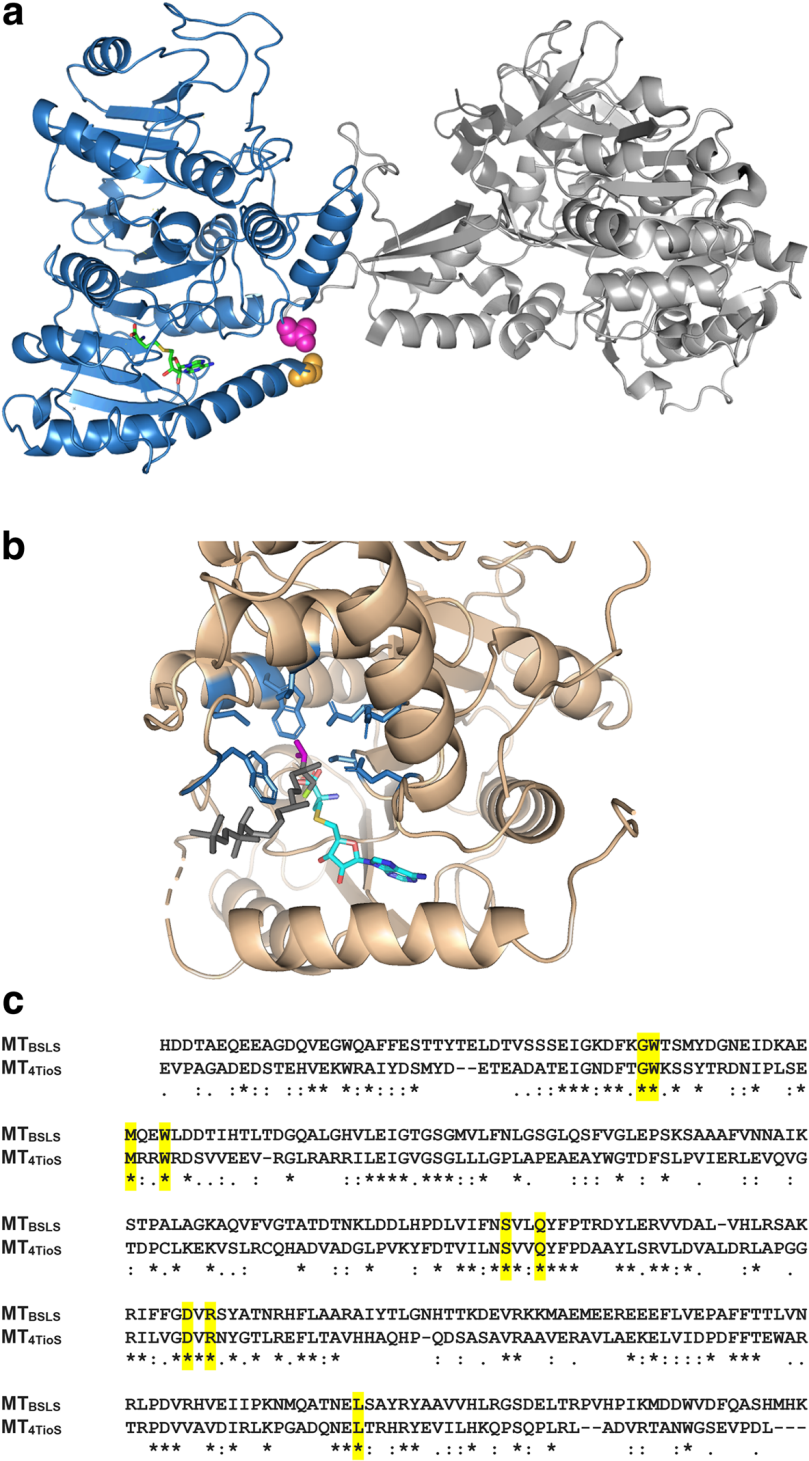
Comparison of the MT domains of TioS and modeled BSLS. **a** A homology model for MT_BSLS_ generated with the Swiss-Model server using the MT-A structure from TioS(A_4a_M_4_A_4b_) (pdb ID 5wmm). Sequence identity between the core of MT_4TioS_ and MT_BSLS_ is 32%. For context purposes, the A domain of TioS is shown in light gray. The generated model of MT_BSLS_ is shown in blue. The active site was identified by aligning the TioS structure (which contained *S*-adenosylhomocysteine, shown in green) with the MT_BSLS_ model. The N- and C-termini of MT_BSLS_ are shown in orange and magenta spheres, respectively. **b** The active site residues of the MT domain in TioS(A_4a_M_4_A_4b_) (pdb ID 5wmm) that are proposed to interact with the valine side chain (magenta) of the enzyme-bound substrate (G525, W526, M540, W543, S632, Q635, D664, R666, and L738, shown in blue). The *N*-methylated product of the reaction (methyl group in green) was modeled into the active site [4]. **c** Residues in TioS(A_4a_M_4_A_4b_) that interact with the substrate amino acid are conserved (yellow) in MT_BSLS_

Intrigued by the observation that the BSLS system produces more NRP with *N*-methyl-L-Leu, but the isolated MT_BSLS_ prefers to methylate L-Phe-SNAC, we tested the ability of the intact MT in BSLS to methylate both L-Phe-SNAC and L-Leu-SNAC at a fixed substrate concentration. Compared to the isolated MT_BSLS_ that methylated Phe-SNAC > 7-fold more than L-Leu-SNAC, the embedded MT in BSLS methylated both amino acid derivatives similarly (Fig. 5). In order to gain more structural insight regarding the potential structural differences that might occur between embedded and isolated MT_BSLS,_ we used the recent crystal structure of an embedded MT domain from the TioS NRPS cluster to create a homology model for MT_BSLS_ (Fig. 6a) [16–20]. The TioS structure includes both the MT (with the product *S*-adenosylhomocysteine bound) and A domains which allowed us to examine the predicted MT active site and how the two domains are connected.

The MT in TioS(A_4a_M_4_A_4b_) *N*-methylates L-Val and norcoronamic acid (NCA), and to a lesser extent L-Ile [4]. On the other hand, MT_BSLS_ preferentially *N*-methylates L-Phe and to a lesser extent L-Leu. Although all five substrates in question are all hydrophobic, we found it surprising that all the residues in MT_4TioS_ that are predicted to interact with the side chain of the amino acid substrate are identical between MT_4TioS_ and MT_BSLS_ (Additional file 1: Fig. 6b, c and Additional file 1: Figure S7). This suggests that characteristics other than side chain identities help to govern substrate specificity of the methyltransferase.

In evaluating the TioS(A_4a_M_4_A_4b_) structure, we noted that the backbone of the N-terminal and C-terminal residues of the MT domain reside within ~ 6 Å of each other, and that the positioning of the termini may be governed by the connection to the A domain. Given that the N-terminus of the MT resides at the end of the helix that acts as the bottom of the methyltransferase active site, we rationalized that altering the distance, conformation, or flexibility between the termini (e.g., in the isolated MT_BSLS_) may affect methyltransferase activity.

**Fig. 7:**
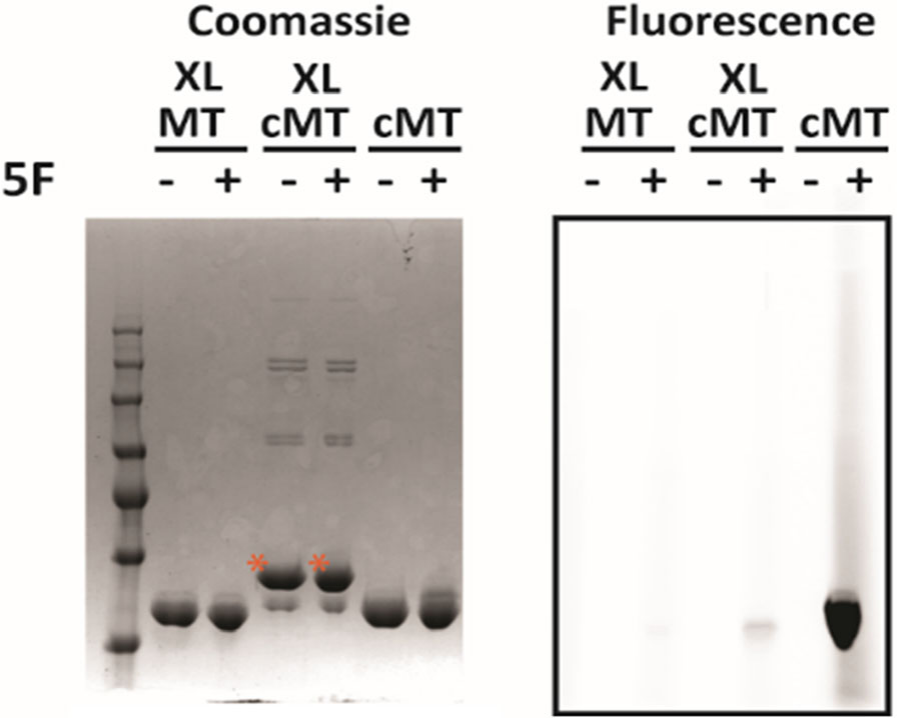
Crosslinking of cMT_BSLS._ Pre-reduced and desalted proteins (20 μM cMT_BSLS_ or MT_BSLS_ as a control) were incubated with 20 μM bis-maleimidoethane or buffer. Free thiol content in the proteins was assessed by quenching samples with 5-fluorosceinyl-maleimide (5F) prior to SDS-PAGE. Gels were imaged for fluorescence and then stained with Coomassie and imaged for total protein. The bands labeled with an asterisk represent crosslinked cMT (XLcMT)

In order to test the theory that increased flexibility or conformation in the N- and C-termini of the isolated MT_BSLS_ affected substrate preference, we designed and purified a construct of MT_BSLS_ that contained an additional cysteine residue at both ends (cMT). We then crosslinked the two cysteine residues using bis-maleimidoethane (BMOE) to create XLcMT (Fig. 7). The BMOE crosslinker has an 8 Å linker, which we hypothesized would fix the two termini at approximately the same distance apart as if the MT was embedded in the A domain. Both cMT and XLcMT were assayed for methyltransferase activity in the presence of either L-Phe-SNAC or L-Leu-SNAC. Crosslinking the N- and C-termini decreased the preference of the methyltransferase for L-Phe over L-Leu (Fig. 5). These data suggest that structural flexibility of the N- and/or C-terminus of MT_BSLS_ plays a role (either by allowing for a different stable conformation of the termini compared to the embedded MT or by providing flexibility at the termini) in the ability of the isolated MT to preferentially methylate L-Phe over L-Leu.

Although XLcMT_BSLS_ displayed less preference for L-Phe-SNAC than MT_BSLS,_ the ratio of Phe:Leu methylated is still higher than that observed for the embedded MT. This could arise from a less than optimal cross-linking distance and incomplete desired crosslinking (~ 85–90%, Fig. 7). We therefore explored how adding a portion of the A domain might influence the ability of the isolated MT domain to differentially methylate L-Phe-SNAC. A construct containing just the small portion of the A domain was designed and purified (aMT_BSLS_) (Figs. 5a and Additional file 1: Figure S8). When assayed in the presence of either L-Phe-SNAC or L-Leu-SNAC, the aMT_BSLS_ construct displayed little preference for L-Phe-SNAC, demonstrating levels of methylation that were similar to the full length BSLS (Fig. 5).

## Discussion

NRPs represent a large family of structurally and functionally diverse natural products. Some of these molecules have shown health-benefiting biological activities and are used as pharmaceuticals, such as the well-known antibiotics penicillin and vancomycin. NRPs are synthesized by modular NRPSs which consist of a series of catalytic domains. The domain structure and specificity of individual domains contribute to the chemical diversity in NRPs. Many different NRPSs, from bacteria or fungi, have been extensively investigated to understand the biosynthetic mechanisms. Based on the mechanistic studies, the enzymes can be engineered to create novel products through genetic approaches, such as domain swapping and active site modification [21]. Some reported NRPSs were found to contain a MT domain that is embedded in an A domain. In this work, we investigated BSLS, an iterative fungal NRPS to understand how embedding of the MT domain in an A domain affects the biosynthetic process and how the substrate specificity of MT is influenced by this unique structural feature.

To probe the role of the MT domain of BSLS in bassianolide biosynthesis, we removed this domain from BSLS. The resulting enzyme BSLS-ΔMT only yielded the nonmethylated product *N*-desmethylbassianolide (Fig. 2a). Similar results were obtained when we inactivated the MT domain in BEAS (Fig. 2b), another fungal NRPS that shares the same domain architecture. Thus, these results revealed that embedding the MT domain is not essential for the assembly of the NRP structures, nor is it necessary for catalysis by other catalytic domains in these modular NRPSs. A previous study on the *N*-MT activities of the cyclosporine synthetase using *N*-methyltransferase inhibitors revealed that a maximum of two desmethyl positions can be tolerated by the enzyme. Further analyses revealed that *N*-methylation of specific amide bond positions in the cyclosporin backbone is required for the downstream domain [22]. This is different from what we observed for BSLS, in which the assembly process can still proceed even if all the three amide bonds are nonmethylated. However, it was found that the yields of demethylated derivatives were much lower than those of bassianolide and beauvericins, suggesting that a methylated derivative of the amino acid precursor is preferred by the C2 and C3 domains for elongation of the depsipeptide chain and final cyclization.

We have previously dissected fungal NRPSs at different positions and found that BSLS and BEAS are quite flexible and co-expression of dissected fragments in the yeast can recover the biosynthesis of bassianolide and beauvericins [11, 15]. Similarly, we also found that the biosynthetic capability of fungal nonreducing PKSs can be restored when the truncated enzymes lacking the product template (PT) domain were co-expressed with the standalone PT in the yeast [23]. However, when we co-expressed BSLS-MT or BEAS-G2131A with standalone MT, no methylated products were detected. Therefore, it can be concluded that natural embedding of a functional MT domain in the A2 domain is required for the synthesis of methylated NRPs.

The MT domain of BSLS was functionally characterized by incubating it with aminoacyl-SNACs in the presence of AdoMet (Fig. 3a), which yielded the corresponding *N*-methylated products. Aminoacyl-SNACs were used to mimic the natural T-tethered substrates. A series of MT constructs, including BSLS, MT_BSLS_, cMT, XLcMT, and aMT, were tested to compare the substrate specificity. The results show that the substrate specificity of MT_BSLS_ is modified by insertion into the A2 domain of BSLS. Among the five constructs, MT_BSLS_ showed a much higher methylation rate with L-Phe-SNAC than with L-Leu-SNAC. cMT showed a lower ratio of methylation of L-Phe-SNAC/L-Leu-SNAC, followed by XLcMT. This suggests that restricting both termini of the isolated MT increases the preference of this domain toward L-Leu-SNAC. aMT possessed a substrate specificity similar to the megaenzyme BSLS, likely due to its structural similarity to the megaenzyme (Additional file 1: Figure S8), further supporting that natural embedding of MT in the A2 domain is critical to narrow down the substrate specificity of the MT domain. Our data suggest that altered substrate specificity is mechanistically achieved by fixing the N- and/or C-terminus of the MT_BSLS_. The somewhat relaxed substrate specificity of the isolated MT_BSLS_ suggests that this MT may be engineered to provide methylation of a variety of amino acids in designer NRPS systems. Embedding the MT could be used to afford selective methylation to the desired amino acid.

Our results also suggest a rationale for why the MT domain in this NRPS is embedded in the A domain instead of positioned as an end-on-end modifying unit or as a separate polypeptide. BSLS synthesizes both bassianolide (Leu-containing) and beauvericins (Phe-containing) in ~ 5:1 ratio when expressed recombinantly in yeast [15]. This indicates that the A domain of BSLS is promiscuous and can activate both L-Leu and L-Phe. However, the MT domain showed a preference for L-Phe-SNAC over L-Leu-SNAC (Fig. 5B) and thus appears to be more promiscuous than the A domain. Embedding the MT tunes the activity of the MT domain against L-Phe, perhaps acting as a secondary gatekeeper. In naturally occurring NRPS systems, it may suggest that MT genes could be swapped/usurped from non-optimal NRPS clusters. Additionally, although the methyl group is not a necessary recognition element for the synthesis of bassianolide/beauvericin (deletion of the MT domain gave rise to a desmethyl product), the fact that we saw no evidence of transmethylation suggests that embedding the MT helps to ensure capture and methylation of the amino acid en route to formation of the NRP product.

## Conclusions

In conclusion, we investigated the role of the MT domain in NRP biosynthesis and how its substrate specificity is affected by protein insertion. The MT domain of BSLS methylates the T-tethered amino acid precursors selected and activated by the A domain. The embedding of a functional MT domain in the A domain is not required for the biosynthesis of the NRP products, but is very critical for the overall efficiency of the assembly line. The substrate specificity of MT is significantly affected by the protein context within which it is present. While A domains are known to be responsible for selecting and activating the biosynthetic precursors for NRPS systems, our results suggest that embedding the MT acts as a secondary gatekeeper for the assembly line. This work thus provides new insights into the embedded MT domain in NRPSs, which will facilitate further engineering of this type of biosynthetic machinery to create structural diversity in natural products.

## Methods

### General methods and materials

Products were analyzed on an Agilent 1200 high performance liquid chromatography (HPLC) instrument. Mass spectra of the compounds were collected by the HPLC coupled with an Agilent 6130 Single Quad mass spectrometer. ^1^H NMR data spectra were acquired on a JEOL instrument (300 MHz). High fidelity DNA polymerase, T4 DNA ligase, restriction enzymes and protein ladder were purchased from New England Biolabs. Other chemicals were purchased from Fisher Scientific. *B. bassiana* ATCC 7159 was obtained from American Type Culture Collection (ATCC). *S. cerevisiae* BJ5464-NpgA (*MAT ura3–52 his3–200 leu2–1 trp1 pep4::HIS3 prb1 1.6R can1 GAL*) was used as a host for heterologous expression, which was a gift from Dr. Nancy Da Silva at the University of California, Irvine. *E. coli* XL1-Blue and RIL were purchased from Stratagene for routine cloning and protein expression, respectively.

### Synthesis of NAC

2.28 g cysteamine hydrochloride (20.0 mmol, 1.0 eq.), 1.12 g KOH (20.0 mmol, 1.0 eq.) and 5.04 g NaHCO_3_ (60.0 mmol, 3.0 eq.) were dissolved in 100 mL of water. To the solution was dropwise added 1.9 mL of acetic anhydride (20.0 mmol, 1.0 eq.). The mixture was stirred at room temperature for 10 min. After adjusting the pH value to 7.3 by concentrated HCl, the resulting mixture was extracted with ethyl acetate (100 mL × 3). The combined organic extract was dried by anhydrous MgSO_4_, filtered and concentrated in vacuo to give *N*-acetylcysteamine (NAC). ^1^H NMR (300 MHz, CDCl_3_): *δ* 5.92 (1H, br s), 3.42 (2H, q, *J* = 8.2 Hz), 2.67 (2H, m), 1.97 (3H, s), 1.35 (1H, t, *J* = 8.2 Hz).

### Synthesis of aminoacyl-SNACs

Each tert-butyloxycarbonyl (Boc)-L-amino acid (1 mmol, 1.0 eq.), N,N′-dicyclohexylcarbodiimide (1 mmol, 1.0 eq.) and hydroxybenzotriazole (1 mmol, 1.0 eq.) were dissolved in 15 mL of trifluoroacetic acid (TFA), followed by adding 106 μL of NAC (1 mmol, 1.0 eq.). After stirring the resulting solution for 45 min at room temperature, 69.1 mg K_2_CO_3_ (0.5 mmol, 0.5 eq.) was added and the reaction mixture stirred for 3 h at room temperature. After filtered, the solvent was removed in vacuo. The residue dissolved in ethyl acetate and washed once with one volume of 10% aqueous NaHCO_3_. The organic layer was dried by anhydrous MgSO_4_, filtered and concentrated in vacuo*.* The crude product was subjected to silica gel column chromatography, eluted with 4% (v/v) MeOH-CHCl_3_, to afford Boc-aminoacyl-SNACs. The Boc group was removed by dissolving the Boc-aminoacyl-SNAC in 50% TFA/CH_2_Cl_2_ and stirring at room temperature for 1 h. After evaporation of the solvents, the residue was taken up in a minimal volume of CH_2_Cl_2_ and precipitated with ether. The resulting solid was washed twice with ether and dried to afford the aminoacyl-SNACs. These synthetic SNAC derivatives were dissolved in deuterated dimethyl sulfoxide (DMSO-*d*_6_) and their ^1^H NMR spectra were collected. The chemical shift (δ) values are given in parts per million (ppm). The coupling constants (*J* values) are presented in hertz (Hz).

Boc-L-Leu-SNAC: ^1^H NMR (300 MHz, DMSO-*d*_6_): *δ* 7.97 (1H, br t, *J* = 5.5 Hz), 7.55 (1H, d, *J* = 7.9 Hz), 4.01 (1H, m), 3.10 (2H, m), 2.83 (2H, m), 1.75 (3H, s), 1.69 (1H, m), 1.59 (1H, m), 1.44 (1H, m), 1.37 (9H, s), 0.83 (3H, d, *J* = 6.5 Hz), 0.79 (3H, d, *J* = 6.5 Hz).

L-Leu-SNAC: ^1^H NMR (300 MHz, DMSO-*d*_6_): *δ* 8.58 (2H, br s), 8.11 (1H, br t, *J* = 5.5 Hz), 4.11 (1H, t, *J* = 6.9 Hz), 3.20 (2H, q, *J* = 6.5 Hz), 3.02 (2H, m), 1.77 (3H, s), 1.75 (1H, m), 1.61 (2H, t, *J* = 7.0 Hz), 0.88 (6H, d, *J* = 6.5 Hz); ESI-MS: [M + H]^+^ *m*/*z* 233.1.

Boc-L-Ile-SNAC: ^1^H NMR (300 MHz, DMSO-*d*_6_): *δ* 7.98 (1H, m), 7.50 (1H, d, *J* = 8.3 Hz), 3.90 (1H, dd, *J* = 7.9, 6.8 Hz), 3.12 (2H, m), 2.83 (2H, m), 1.75 (3H, s), 1.71 (1H, m), 1.52 (1H, m), 1.37 (9H, s), 1.18 (1H, m), 0.80 (3H, d, *J* = 6.8 Hz), 0.77 (3H, t, *J* = 7.0 Hz).

L-Ile-SNAC: ^1^H NMR (300 MHz, DMSO-*d*_6_): *δ* 8.42 (2H, br s), 8.06 (1H, br t, *J* = 5.5 Hz), 4.16 (1H, d, *J* = 4.1 Hz), 3.20 (2H, m), 3.02 (2H, m), 1.87 (1H, m), 1.76 (3H, s), 1.43 (1H, m), 1.22 (1H, m), 0.91 (3H, d, *J* = 6.9 Hz), 0.86 (3H, t, *J* = 7.4 Hz); ESI-MS: [M + H]^+^ *m*/*z* 233.1.

Boc-L-Val-SNAC: ^1^H NMR (300 MHz, DMSO-*d*_6_): *δ* 7.98 (1H, m), 7.49 (1H, d, *J* = 8.2 Hz), 3.87 (1H, dd, *J* = 8.3, 6.5 Hz), 3.12 (2H, m), 2.83 (2H, m), 2.04 (1H, m), 1.75 (3H, s), 1.38 (9H, s), 0.83 (6H, d, *J* = 6.9 Hz).

L-Val-SNAC: ^1^H NMR (300 MHz, DMSO-*d*_6_): *δ* 8.43 (2H, br s), 8.07 (1H, br t, *J* = 5.1 Hz), 4.12 (1H, d, *J* = 4.8 Hz), 3.20 (2H, m), 3.02 (2H, m), 2.16 (1H, m), 1.76 (3H, s), 0.96 (3H, d, *J* = 6.9 Hz), 0.92 (3H, d, *J* = 7.2 Hz). ESI-MS: [M + H]^+^ *m*/*z* 219.1.

Boc-L-Phe-SNAC: ^1^H NMR (300 MHz, DMSO-*d*_6_): *δ* 8.02 (1H, m), 7.62 (1H, d, *J* = 8.2 Hz), 7.22 (5H, m), 4.21 (1H, m), 3.13 (2H, m), 3.03 (1H, dd, *J* = 14.2, 4.3 Hz), 2.84 (2H, m), 2.76 (1H, dd, *J* = 14.2, 10.6 Hz), 1.76 (3H, s), 1.29 (9H, s).

L-Phe-SNAC: ^1^H NMR (300 MHz, DMSO-*d*_6_): *δ* 8.52 (2H, m), 8.01 (1H, t, *J* = 5.2 Hz), 7.25 (5H, m), 4.47 (1H, t, *J* = 6.9 Hz), 3.10 (4H, m), 2.95 (2H, m), 1.77 (3H, s). ESI-MS: [M + H]^+^ *m*/*z* 267.2.

### Construction of expression plasmids

The cloning vector pJET1.2 (ThermoFisher Scientific) was used for general cloning. pET28a (Novagen) was used for protein expression in *E. coli*. The *E. coli/S. cerevisiae* shuttle vectors YEpADH2p-URA3 and YEpADH2p-TRP1 were used for expression or co-expression experiments in *S. cerevisiae* BJ5464-NpgA.

To construct BSLS-ΔMT in a yeast expression vector, we used pDY42 [12] as the starting plasmid. Our cloning strategy was to work on the fragment from the Bsu36I site (5518) to the C-terminal end (9441). Two pairs of primers, BSLS-Bsu36I5518-F/BSLS-without-MT-R and BSLS-without-MT-F/2nd-BSLS-B-R-PmlI (Table 1), were used to perform SOE PCR to amplify a fragment without the MT domain, which was ligated to pJET1.2 to yield pDY254 (Table 2). After the plasmid was verified by sequencing, this fragment was excised by Bsu36I and PmlI and used to replace the original fragment from the 5518 to the end of *bsls* in pDY42 between the same sites, yielding pDY265 (Table 2).

**Table 1:** Primers used in this study

Primer	Sequence	Restriction sites
BEAS-S2131A-F	5′-CCTGGAGATTGGAACCGCTACAGGTATGATCTTGTT-3′	None
BEAS-S2131A-R	5′-ACATGCCCCGGTACATGACCGTCGCGTAGAGTAT-3′	None
BSLS-Bsu36I5518-F	5′-AACCTCAGGATGCTGTCGATGCG-3′	Bsu36I
BSLS-without-MT-R	5′-CACCGCCACACGTCGCTTTTCCGCAACCACAAATCCG-3′	None
BSLS-without-MT-F	5′-CGGATTTGTGGTTGCGGAAAAGCGACGTGTGGCGGTG-3′	None
2nd-BSLS-B-R-PmlI	5′-AACACGTGTAAAGACGCATTCAAAGCCT-3′	PmlI
MT_BSLS_-F-NheI	5′-AAGCTAGCCACGACGACACTGCCGAACA-3′	NheI
MT_BSLS_-R-EcoRI	5′-AAGAATTCTCACTGAAGTCGCTGGAGAGGTT-3′	EcoRI
MT_BSLS_-F-NdeI	5′-AACATATGCACGACGACACTGCCGAACA-3′	NdeI
MT_BSLS_-R-PmeI	5′-AAGTTTAAACTCACTGAAGTCGCTGGAGAGGTT-3′	PmeI
MT_BEAS_-F-NdeI	5′-AACATATGGCTGACGATGCCGTTGAGCA-3′	NdeI
MT_BEAS_-R-PmeI	5′-AAGTTTAAACCTGCAGCCGCTGCAGCGGCC-3′	PmeI
aMT_BSLS_-F-SpeI	5′-AAACTAGTCAATTCAAGATTCGAAGTAACCGCATC-3′	SpeI
aMT_BSLS_-R-EcoRI	5′-AAAGAATTCTCACGCCACAATGTGGGAAG-3′	EcoRI
cMT_BSLS_-F-NheI	5′- AAAGCTAGCTGCGTTGCGGAACACG-3′	NheI
cMT_BSLS_-R-EcoRI	5′-TTTGAATTCTCAGCACTGGAGAGGTTGATTGGTGAG-3′	EcoRI

**Table 2:** Plasmids used in this study

Plasmid	Description	Restriction sites	Source
pDY37	*beas* in YEpADH2p-URA3	NheI and PmlI	[12]
pDY42	*bsls* in YEpADH2p-URA3	NdeI and PmlI	[12]
pDY170	*beas-G2131A* in YEpADH2p-URA3	NheI and PmlI	This work
pDY254	*bsls*_*(5818–9438*_*)-Δmt* in pJET1.2	Bsu36I and PmlI	This work
pDY265	*bsls-Δmt in* YEpADH2p-URA3	Bsu36I and PmlI	This work
pDY267	MT_BEAS_ in pJET1.2	NdeI and PmeI	This work
pDY268	MT_BEAS_ in YEpADH2p-TRP1	NdeI and PmeI	This work
pFC30	MT_BSLS_ in pJET1.2	NdeI and PmeI	This work
pFC31	MT_BSLS_ in YEpADH2p-TRP1	NdeI and PmeI	This work
pZJ135	MT_BSLS_ in pJET1.2	NheI and EcoRI	This work
pJCZ22	MT_BSLS_ in pET28a	NheI and EcoRI	This work
paMTBSLS	aMT_BSLS_ in pET28a (a small portion of the A_2_ domain+MT of BSLS)	NheI and EcoRI	This work
pcMTBSLS	cMT_BSLS_ in pET28a (MT of BSLS containing an additional cysteine residue at both the N- and C-termini of the MT domain)	NheI and EcoRI	This work

The YEpADH2p-URA3 derived *beas*-harboring plasmid pDY37 [12] was used as the template for PCR with a pair of primers, BEAS-S2131A-F and BEAS-S2131A-R (Table 1). The mutated nucleotide was included in BEAS-S2131A-F. The PCR product was treated with DpnI at 37 °C for 24 h to digest the template. T4 DNA ligase was then added to ligate the PCR product, which was then introduced into *E. coli* XL-Blue through chemical transformation. Correct clones were selected by ampicillin resistance and confirmed by digestion check and sequencing. The correct BEAS-G2131A plasmid was named pDY170 (Table 2).

The gene encoding the MT domain of BSLS was amplified via PCR from the genomic DNA of *B. bassiana* using primers MT_BSLS_-F-NheI and MT_BSLS_-R-EcoRI (Table 1). The PCR product was ligated into pJET1.2 and introduced into *E. coli* XL1-Blue to afford pZJ135 (Table 2). pZJ135 was digested with NheI and EcoRI, and the fragment was ligated into pET28a between the corresponding restriction sites to yield pJCZ22 (Table 2). The same MT domain was amplified with MT_BSLS_-F-NdeI and MT_BSLS_-R-PmeI and ligated into pJET1.2 to yield pFC30 (Table 2). The gene fragment was then excised using NdeI and PmeI and ligated into YEpADH2p-TRP1 between the same sites to yield pFC31 (Table 2). Similarly, the MT domain of BEAS was amplified with MT_BEAS_-F-NdeI and MT_BEAS_-R-PmeI and ligated into pJET1.2 to afford pDY267 (Table 2). The MT_BEAS_ gene fragment was excised from pDY267 using NdeI and PmeI and ligated into YEpADH2p-TRP1 between the same sites to yield pDY268 (Table 2).

The gene for the aMT_BSLS_ construct, which also contains the small fragment of the A2 domain, was amplified using primers aMT_BSLS_-F-SpeI and aMT_BSLS_-R-EcoRI (Table 1). The digested PCR product was ligated into NheI/EcoRI digested pET28a to yield paMTBSLS (Table 2). The gene for the cMT_BSLS_ construct, which contains an additional cysteine residue at both the N- and C-termini, was amplified using primers cMT_BSLS_-F-NheI and cMT_BSLS_-R-EcoRI. The digested PCR product was ligated into pET28a between the NheI and EcoRI sites to yield pcMTBSLS (Table 2). All plasmids were verified by DNA sequencing.

### Expression and purification of MT constructs

The 348 kDa BSLS was expressed in *S. cerevisiae* BJ5464-NpgA/pDY42 (Table 2) and purified using Ni-NTA chromatography as described in our previous work [11]. To purify MT_BSLS_, pJCZ22 was introduced into *E. coli* RIL. The resulting strain was grown at 37 °C in Luria-Bertani (LB) medium supplemented with 35 μg mL^− 1^ kanamycin and 25 μg mL^− 1^ chloramphenical to an OD_600_ of 0.4–0.6, and induced by 200 μM isopropyl-1-thio-β-D-galactoside (IPTG) for 16 h at 28 °C. The cells were harvested by centrifugation at 3,500 rpm for 10 min, re-suspended in 30 mL of cold lysis buffer [20 mM Tris-HCl (pH 7.9), 0.5 M NaCl, pH 7.9] and sonicated on ice. Cellular debris was removed by centrifugation at 20,000 rpm for 30 min at 4 °C. Ni-NTA agarose resin (Qiagen) was added to the supernatant (4 mL L^− 1^ of culture) and the mixture was shaken at 4 °C for 4 h to ensure the His_6_-tagged protein was well absorbed. The protein resin mixture was loaded into a gravity flow column and protein was purified with an increasing concentration of imidazole in buffer A [50 mM Tris-HCl, pH 7.9, 2 mM ethylenediaminetetraacetic acid (EDTA), 1 mM dithiothreitol (DTT) and 0.2 mM phenylmethylsulfonyl fluoride (PMSF)]. The purified MT_BSLS_ was concentrated and washed with 50 mM Tris-HCl buffer (pH 7.9) by Centriprep filter devices (Amicon Inc.).

The cMT_BSLS_ and aMT_BSLS_ proteins were expressed in *E. coli* RIL cells with 200 μM IPTG for 24 h at 16 °C. Cells were resuspended in the lysis buffer, lysed by sonication, and centrifuged as noted above. Clarified lysate was incubated with Goldbio Nickel resin (0.5 mL resin/g of cells) for 4 h while shaking at 4 °C. The nickel resin was batch-washed and the desired proteins eluted with a stepped imidazole gradient. An additional anion exchange step (MonoQ) was required to purify the aMT_BSLS_ construct. Protein purity was evaluated by sodium dodecyl sulfate polyacrylamide gel electrophoresis (SDS-PAGE). Selected fractions were pooled and concentrated in a 30 K MWCO Millipore spin concentrator that was also used to exchange the buffer (50 mM Tris-HCl, 150 mM NaCl, 2 mM β-mercaptoethanol, 5% glycerol, pH 7.5). Proteins were frozen in liquid nitrogen and stored at − 80 °C.

### Production of *N*-desmethylated NRPs using engineered yeast strains

pDY265 and pDY170 were respectively transferred into *S. cerevisiae* BJ5464-NpgA. The strains were grown in SC-URA medium as described in our previous work [12]. The products were extracted with ethyl acetate and analyzed by LC-MS using a Zorbax SB-C18 reversed-phase column (5 μm, 150 mm × 4.6 mm) at 210 nm, washed with a gradient of methanol-water (80 to 100% over 20 min) at a flow rate of 1 mL min^− 1^. The demethylated derivative of beauvericin was isolated by HPLC from 1 L of culture of *S. cerevisiae* BJ5464-NpgA/pDY170 for NMR analysis (Additional file 1: Figures S2-S4).

For co-expression of an isolated MT domain with BSLS-ΔMT or BEAS-G2121A, pFC31 and pDY265, or pDY268 and pDY170, were co-transferred into *S. cerevisiae* BJ5464-NpgA. The strains were grown in SC-URA-TRP medium, and the products were extracted and analyzed as described above.

### LC-MS identification of methylated products of aminoacyl SNACs

A typical methylation assay mixture (100 μL) consisted of 6.4 μM MT_BSLS_, 0.8 mM aminoacyl-SNAC, and 2.4 mM AdoMet in 100 mM Tris-HCl buffer (pH 7.5). The reaction mixtures were incubated at 25 °C for 30 min and then quenched with MeOH (50 μL). Substrate controls included all the components except MT_BSLS_. The mixtures were briefly vortexed and centrifuged at 15,000 rpm for 5 min to remove the precipitated protein before the samples were injected into LC-MS for analysis. The supernatants were analyzed on an Agilent Single Quad LC-MS by using a Zorbax SB-C18 reversed-phase column (5 μm, 150 mm × 4.6 mm) at 235 nm, eluted with a solvent gradient of increasing acetonitrile (10–15%) in H_2_O containing 0.1% trifluoroacetic acid with a flow rate of 1 mL min^− 1^.

### Quantification of the activity of the MT domains

Methyltransferase activity was quantified using a radiometric approach. Methylation reactions were conducted in 50 mM Na_2_HPO_4_, [pH 7.5], 100 μM AdoMet (99 μM unlabeled AdoMet and 1 μM *S*-[methyl-^3^H]-AdoMet (PerkinElmer Life Sciences; stock solution of 5–10 mM (~ 75 Ci/mmol) in 10 mM H_2_SO_4_/EtOH (9,1, v/v)) and 1.5 μM enzyme in a total volume of 25 μL. The reactions were initiated with amino acyl-SNAC substrates at varying concentrations as indicated in the figures and incubated at 25 °C. Aliquots (10 μL) at various times were removed from the reaction and placed in 20 μL of 1 M Tris-HCl buffer (pH 9.8) and immediately diluted by the addition of 200 μL of H_2_O. The *N*-methylated product was extracted using 400 μL of ethyl acetate. Half the organic layer was added to 4.8 mL of scintillation cocktail and the radioactivity present quantified using scintillation counting.

### Crosslinking of methyltransferases

Proteins undergoing crosslinking were first pre-reduced with 1 mM DTT for 20 min and rapidly desalted using Zebaspin columns (Pierce). Crosslinking was accomplished by incubating 20 μM protein (cMT_BSLS_, or MT_BSLS_ as a control) with 20 μM bis-maleimidoethane (BMOE) on ice for 1 h. For gel analyses, samples were either treated with 5-fluorosceinyl-maleimide (5F) in SDS sample buffer lacking β-mercaptoethanol for 10 min, followed by a DTT quench step, or were treated with SDS sample buffer containing β-mercaptoethanol for 10 min, followed by a DTT quench step. SDS-gels were first imaged for fluorescence resulting from the covalent addition of 5F to free thiols and then stained with Coommassie. The per cent of desired crosslinking (noted by the asterisk in Fig. 7) was estimated at 85–90% by taking the intensity of the desired band (measured by densitometry) divided by the total intensity of all bands in the lane. For crosslinking prior to activity measurements, cMT_BSLS_ was pre-reduced and incubated in the presence and absence (control) of the BMOE crosslinker as described above. After 1 h the reactions were quenched with 2 mM DTT and desalted using Zebaspin columns.

## Additional file

Additional file 1: **Figure S1.** ESI-MS (+) spectra of the demethylated nonribosomal peptides. (A) *N*-Desmethylbassianolide. (B) *N*-Desmethylbeauvericin. (C) *N*-Desmethylbeauvericin A. (D) *N*-Desmethylbeauvericin B. **Figure S2.** ^1^H NMR spectrum of *N*-desmethylbeauvericin. **Figure S3.** ^13^C NMR spectrum of *N*-desmethylbeauvericin. **Figure S4.** Selected ^1^H-^1^H COSY and HMBC corrections for *N*-desmethylbeauvericin. **Figure S5.** HPLC analysis (210 nm) of products from the co-expression of isolated MT domain with MT-removed BSLS/MT-inactivated BEAS in *S. cerevisiae*. (A) Co-expression of MT_(BSLS)_ with BSLS-ΔMT. (B) Co-expression of MT_(BEAS)_ with BEAS-G2131A. **Figure S6.** ESI-MS(+) spectrum of the *N*-methylated products of L-Ile-SNAC (A) and L-Val-SNAC. **Figure S7.** Location of residues in MT_BSLS_ that are proposed to interact with the amino acid substrate. The active site residues (red) of the MT domain in TioS(A_4a_M_4_A_4b_) (pdb ID 5wmm) (gray) that are proposed to interact with the valine side chain (magenta) of the enzyme-bound substrate (G525, W526, M540, W543, S632, Q635, D664, R666, and L738) were mapped onto the homology model of MT_BSLS_ (blue, with limon amino acid side chains). AdoHcy is shown in green and the tethered substrate for TioS(A_4a_M_4_A_4b_) is shown in dark gray. **Figure S8.** Architecture of the aMT construct. Adenylation domains consist of one polypeptide containing a large subunit (light gray) and a small subunit (dark gray). The active site exists between the two subunits. The “aMT” construct used in these studies contains the entire MT domain and the small subunit of the adenylation domain. Figure 6a was used in the preparation of this figure. (DOCX 1951 kb)

## Data Availability

Data and materials are available from the corresponding authors upon reasonable request.

## Abbreviations

5F: 5-Fluorosceinyl-maleimide
A: Adenylation
AdoMet: *S*-Adenosyl methionine
Aminoacyl-SNACs: Aminoacyl-*N*-acetylcysteamine thioesters
BEAS: Beauvericin synthetase
BMOE: Bis-Maleimidoethane
Boc: Tert-Butyloxycarbonyl
BSLS: Bassianolide synthetase
C: Condensation
D-Hiv: D-2-Hydroxyisovalerate
D-Hmv: D-2-Hydroxy-3-methylvalerate
DTT: Dithiothreitol
E: Epimerization
EDTA: Ethylenediaminetetraacetic acid
HPLC: High performance liquid chromatography
IPTG: Isopropyl-1-thio-β-D-galactoside
MS: Mass spectrometry
MT: Methyltransferase
NAC: *N*-Acetylcysteamine
NCA: Norcoronamic acid
NRP: Nonribosomal peptide
NRPS: Nonribosomal peptide synthetase
PKS: Polyketide synthase
PMSF: Phenylmethylsulfonyl fluoride
SDS-PAGE: Sodium dodecyl sulfate polyacrylamide gel electrophoresis
T: Thiolation
TE: Thioesterase
TFA: Trifluoroacetic acid
