# Supplementary material for: Modified substrate specificity of a methyltransferase domain by protein insertion into an adenylation domain of the bassianolide synthetase
Source: J Biol Eng. 2019 Jul 31;13:65. doi: 10.1186/s13036-019-0195-y (PMC6670151; doi:10.1186/s13036-019-0195-y)
Supplement: Supplementary file 1 — Figure S1. ESI-MS (+) spectra of the demethylated nonribosomal peptides. (A) N-Desmethylbassianolide. (B) N-Desmethylbeauvericin. (C) N-Desmethylbeauvericin A. (D) N-Desmethylbeauvericin B. Figure S2. 1H NMR spectrum of N-desmethylbeauvericin. Figure S3. 13C NMR spectrum of N-desmethylbeauvericin. Figure S4. Selected 1H-1H COSY and HMBC corrections for N-desmethylbeauvericin. Figure S5. HPLC analysis (210 nm) of products from the co-expression of isolated MT domain with MT-removed BSLS/MT-inactivated BEAS in S. cerevisiae. (A) Co-expression of MT(BSLS) with BSLS-ΔMT. (B) Co-expression of MT(BEAS) with BEAS-G2131A. Figure S6. ESI-MS(+) spectrum of the N-methylated products of L-Ile-SNAC (A) and L-Val-SNAC. Figure S7. Location of residues in MTBSLS that are proposed to interact with the amino acid substrate. The active site residues (red) of the MT domain in TioS(A4aM4A4b) (pdb ID 5wmm) (gray) that are proposed to interact with the valine side chain (magenta) of the enzyme-bound substrate (G525, W526, M540, W543, S632, Q635, D664, R666, and L738) were mapped onto the homology model of MTBSLS (blue, with limon amino acid side chains). AdoHcy is shown in green and the tethered substrate for TioS(A4aM4A4b) is shown in dark gray. Figure S8. Architecture of the aMT construct. Adenylation domains consist of one polypeptide containing a large subunit (light gray) and a small subunit (dark gray). The active site exists between the two subunits. The “aMT” construct used in these studies contains the entire MT domain and the small subunit of the adenylation domain. Figure 6a was used in the preparation of this figure. (DOCX 1951 kb) [file 13036_2019_195_MOESM1_ESM.docx]

**Supporting Information**

**for**

**Modified substrate specificity of a methyltransferase domain by protein insertion into an adenylation domain of the bassianolide synthetase**

Fuchao Xu,^1^ Russell Butler,^2^ Kyle May,^2^ Megi Rexhepaj,^2^ Dayu Yu,^1,3^, Jiachen Zi,^1^ Yi Chen,^1^ Yonghong Liang,^1^ Jia Zeng,^1^ Joan Hevel,^2^* Jixun Zhan^1^*

1. Department of Biological Engineering, Utah State University, 4105 Old Main Hill, Logan, UT 84322-4105, USA
2. Department of Chemistry and Biochemistry, Utah State University, 0300 Old Main Hill, Logan, UT 84322-0300, USA
3. Department of Applied Chemistry and Biological Engineering, College of Chemical Engineering, Northeast Electric Power University, Jilin, Jilin 132012, China

* **Correspondence:** [joanie.hevel@usu.edu](mailto:joanie.hevel@usu.edu) (J. Hevel) and [jixun.zhan@usu.edu](mailto:jixun.zhan@usu.edu) (J. Zhan).

| A | B |
| --- | --- |
|  |  |
| C | D |
|  |  |

**Additional file 1: Figure S1.** ESI-MS (+) spectra of the demethylated nonribosomal peptides. (A) *N*-Desmethylbassianolide. (B) *N*-Desmethylbeauvericin. (C) *N*-Desmethylbeauvericin A. (D) *N*-Desmethylbeauvericin B.

**Additional file 1: Figure S2.** ^1^H NMR spectrum of *N*-desmethylbeauvericin.

**Additional file 1: Figure S3.** ^13^C NMR spectrum of *N*-desmethylbeauvericin.

**Figure S4.** Selected ^1^H-^1^H COSY and HMBC corrections for *N*-desmethylbeauvericin.

| A | B |
| --- | --- |
|  |  |

**Additional file 1: Figure S5.** HPLC analysis (210 nm) of products from the co-expression of isolated MT domain with MT-removed BSLS/MT-inactivated BEAS in *S. cerevisiae*. (A) Co-expression of MT_(BSLS)_ with BSLS-ΔMT. (B) Co-expression of MT_(BEAS)_ with BEAS-G2131A.

| A | B |
| --- | --- |
|  |  |

**Additional file 1: Figure S6.** ESI-MS(+) spectrum of the *N*-methylated products of L-Ile-SNAC (A) and L-Val-SNAC.


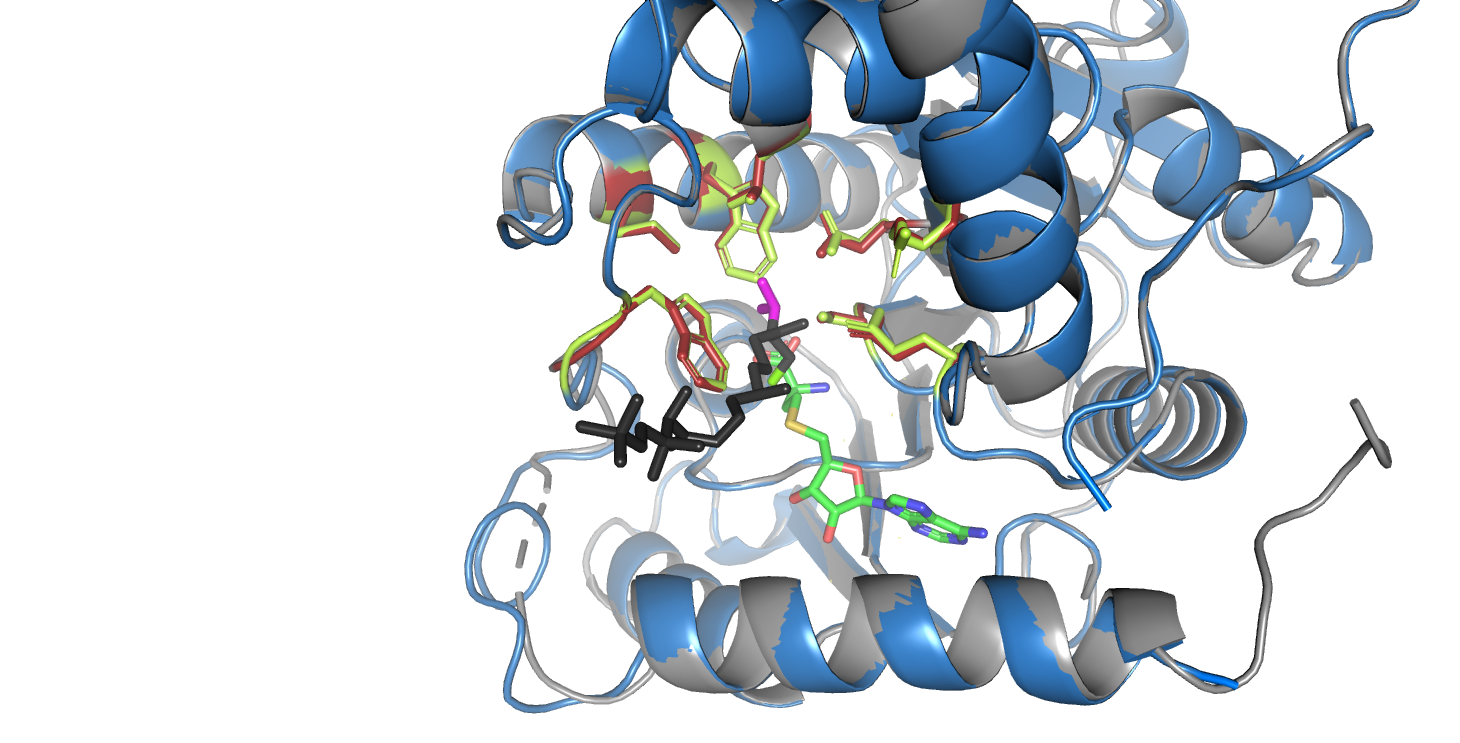


**Additional file 1: Figure S7.** Location of residues in MT_BSLS_ that are proposed to interact with the amino acid substrate. The active site residues (red) of the MT domain in TioS(A_4a_M_4_A_4b_) (pdb ID 5wmm) (gray) that are proposed to interact with the valine side chain (magenta) of the enzyme-bound substrate (G525, W526, M540, W543, S632, Q635, D664, R666, and L738) were mapped onto the homology model of MT_BSLS_ (blue, with limon amino acid side chains). AdoHcy is shown in green and the tethered substrate for TioS(A_4a_M_4_A_4b_) is shown in dark gray.

**
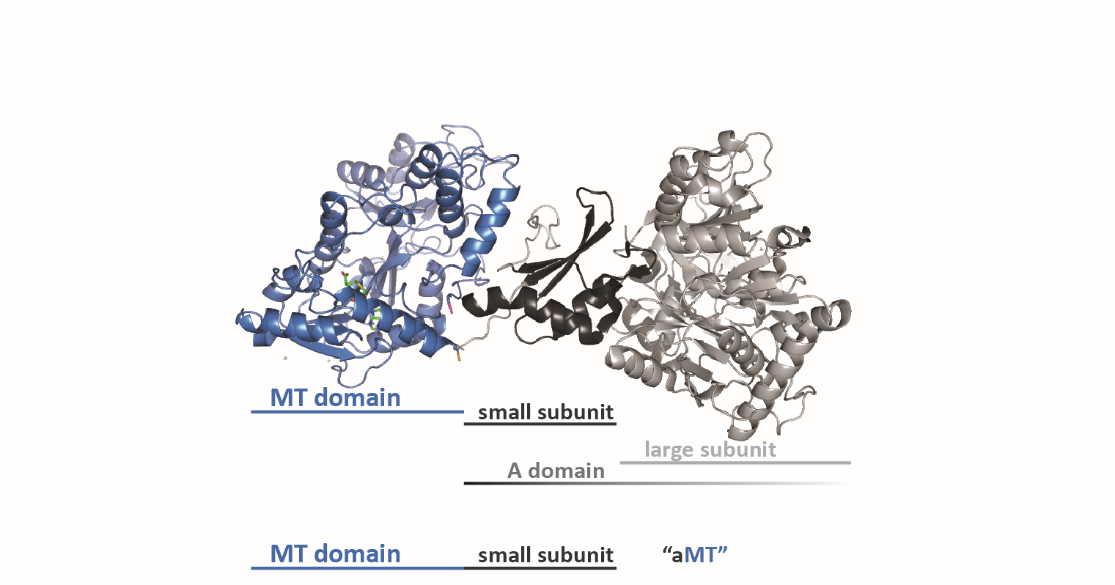
**

**Additional file 1: Figure S8.** Architecture of the aMT construct. Adenylation domains consist of one polypeptide containing a large subunit (light gray) and a small subunit (dark gray). The active site exists between the two subunits. The “aMT” construct used in these studies contains the entire MT domain and the small subunit of the adenylation domain. Fig. 6A was used in the preparation of this figure.
